# Disturbed Amino Acid Metabolism in HIV: Association with Neuropsychiatric Symptoms

**DOI:** 10.3389/fpsyt.2015.00097

**Published:** 2015-07-14

**Authors:** Johanna M. Gostner, Kathrin Becker, Katharina Kurz, Dietmar Fuchs

**Affiliations:** ^1^Division of Medical Biochemistry, Biocenter, Medical University of Innsbruck, Innsbruck, Austria; ^2^Division of Biological Chemistry, Biocenter, Medical University of Innsbruck, Innsbruck, Austria; ^3^Department of Internal Medicine VI, Medical University of Innsbruck, Innsbruck, Austria

**Keywords:** HIV, tryptophan, kynurenine, indoleamine 2,3-dioxygenase, phenylalanine, tyrosine, phenylalanine 4-hydroxylase, tetrahydrobiopterin

## Abstract

Blood levels of the amino acid phenylalanine, as well as of the tryptophan breakdown product kynurenine, are found to be elevated in human immunodeficiency virus type 1 (HIV-1)-infected patients. Both essential amino acids, tryptophan and phenylalanine, are important precursor molecules for neurotransmitter biosynthesis. Thus, dysregulated amino acid metabolism may be related to disease-associated neuropsychiatric symptoms, such as development of depression, fatigue, and cognitive impairment. Increased phenylalanine/tyrosine and kynurenine/tryptophan ratios are associated with immune activation in patients with HIV-1 infection and decrease upon effective antiretroviral therapy. Recent large-scale metabolic studies have confirmed the crucial involvement of tryptophan and phenylalanine metabolism in HIV-associated disease. Herein, we summarize the current status of the role of tryptophan and phenylalanine metabolism in HIV disease and discuss how inflammatory stress-associated dysregulation of amino acid metabolism may be part of the pathophysiology of common HIV-associated neuropsychiatric conditions.

## Introduction

Human immunodeficiency virus type 1 (HIV-1) infection is associated with neuropsychopathologic disturbances that range from behavioral changes and mild cognitive and motor impairments to depression and severe mental problems as reported in patients with manifested AIDS dementia complex (1). These conditions are comprehensively termed HIV-associated neurocognitive disorders (HAND). Besides the presence and severity of neurocognitive impairment and functional decline, the definitional criteria for HAND also comprise a variety of neuropsychiatric relevant comorbid conditions (2). Steady deterioration of neurocognitive performance in patients not only impairs their quality of life but is also associated with an increased mortality rate. Although it has been reported that effective antiretroviral therapy (ART) can partially preserve or even improve neurological function and decrease morbidity and mortality (3), neurocognitive disturbances are still highly prevalent in HIV patients receiving treatment (4). The development of depressive symptoms is the most frequent manifestation (5–8). A number of psychosocial aspects, in addition to pathophysiological factors, may contribute to the high rate of depressive illness in these patients (7).

The molecular basis of HAND- and HIV-associated depressive symptoms still remains to be elucidated in detail (9). The incomplete clearance of viral load owing to the poor accessibility of the central nervous system to antiretroviral drugs or the development of resistant virus strains during therapy is suspected to be of importance (10). In addition, neuronal damage may arise from toxic viral products or from activated brain macrophages and microglia releasing inflammatory mediators (9, 11). Increasing evidence suggests that changes in amino acid metabolism occur as a result of these viral and inflammatory insults during infection, and that these metabolic changes may play a critical role in HAND. This review will cover recent advances in the field of inflammatory stress-associated dysregulation of amino acid metabolism in HIV and its association with neuropsychiatric symptoms.

## Metabolic Changes in HIV Disease

HIV disease is characterized by severe metabolic changes. Anorexia, malabsorption, increased resting energy expenditure, and specific disturbances in protein turnover are common in HIV and AIDS patients (12, 13). However, not only HIV infection itself but also effective treatment is associated with the development of severe metabolic disorders, such as insulin resistance, diabetes mellitus (14), and lipodystrophy syndrome (15).

The immune response and metabolic pathways are highly cross-regulated (16). Also, depression and cognitive impairment are closely linked to chronic inflammation. Changes in amino acid metabolism and neurotransmitter synthesis play a major role in the etiology of such conditions (17, 18) and constitute potential intervention points for therapeutic strategies. In this regard, phenylalanine (Phe) and tryptophan (Trp) metabolism are among the most intensively discussed pathways in the literature (18, 19).

Diminished breakdown of Phe to tyrosine (Tyr) (20) and the accelerated conversion of Trp to kynurenine (Kyn) (21) were shown to correlate with elevated levels of immune activation markers, e.g., neopterin or interferon-γ (IFN-γ), in HIV-infected individuals (22, 23). Trp and Tyr are precursor molecules for serotonin and dopaminergic neurotransmitters, respectively. Thus, disturbed metabolism can negatively affect neuropsychoimmunological circuits (24) and can contribute to the pathophysiology of common HIV-associated neuropsychiatric symptoms.

Phenylalanine is converted to Tyr via the tetrahydrobiopterin (BH_4_)-dependent enzyme phenylalanine 4-hydroxylase. Phe turnover is reduced in chronic inflammatory conditions, consequently affecting dopamine, adrenaline, and noradrenaline synthesis, as tyrosine is the precursor of these neurotransmitters. Also, maintenance of adequate levels of tryptophan is essential, as tryptophan hydroxylation via the BH_4_-dependent enzyme tryptophan 5-hydroxylase is the rate limiting step for 5-hydroxytryptamine (serotonin) formation. During inflammation, peripheral Trp levels are predominantly dependent on the activity of enzyme (IDO1), which is activated mainly via the T helper (Th) type 1 cytokine IFN-γ.

**Figure 1:**
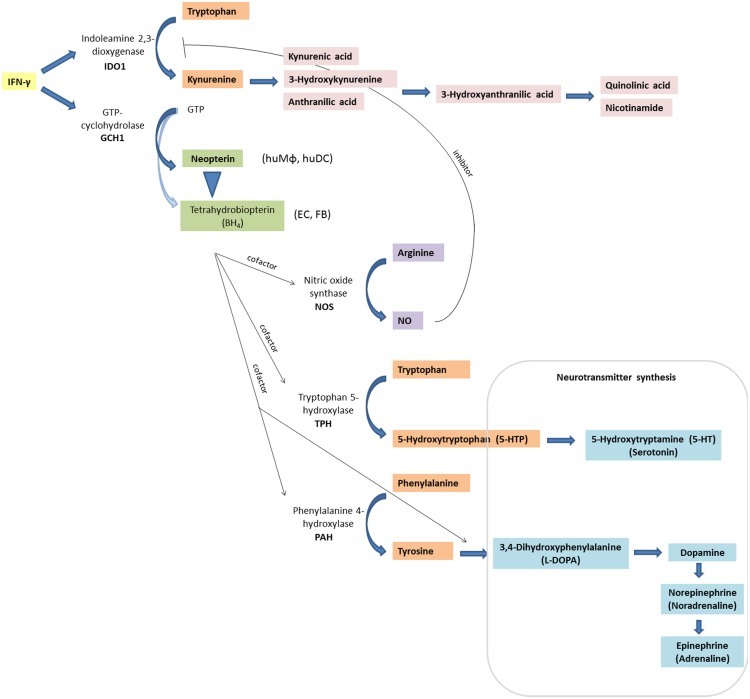
**Inflammation-associated biochemical pathways: interferon-γ (IFN-γ) signaling activates enzymes indoleamine 2,3-dioxygenase (IDO1) and GTP-cyclohydrolase (GCH1), which convert tryptophan to kynurenine, and GTP to neopterin or tetrahydrobioterin (BH_4_), respectively**. In contrast to other cell types and macrophages of other species, human macrophages (huMΦ) produce predominantly neopterin (EC, endothelial cells; FB, fibroblasts). Also, BH_4_ is sensitive to oxidation, thus levels decrease under oxidative stress conditions. Absence of BH_4_, an important cofactor for several monooxygenase, impairs the function of the nitric oxide (NO)-producing enzyme inducible nitric oxide synthase (iNOS), and of tryptophan 5-hydroxylase (TPH), which is involved in serotonin synthesis. Also, phenylalanine 4-hydroxylase (PAH) and aromatic l-amino acid decarboxylase, which are both involved in catecholamine synthesis, need BH_4_ as a cofactor.

In the following, relevant inflammatory stress-related pathways (Figure 1) will be introduced followed by describing their alteration in HIV disease in patients on and off therapy.

## Tryptophan Metabolism

Breakdown of Trp into Kyn is a crucial biochemical pathway within the biosynthesis of nicotinamide adenosine dinucleotides (NAD/NADH) that are required cofactors for many enzymes. The pathway is also important for the functioning of immune responses because during immune activation, Th1 cytokines, most importantly, IFN-γ, induce the expression and activation of enzyme IDO1 (EC:1.13.11.52) in human monocytes/macrophages and dendritic cells (25). In addition, other cell types, such as endothelial, epithelial cells, and fibroblasts, are able to activate IDO1 in response to inflammatory stimuli. Deprivation of this essential amino acid not only inhibits pathogen growth, but also slows down T-cell responses, thereby being a regulatory feedback mechanism that protects the immune system from overreactions (26). IDO1 activation is associated with the generation of a regulatory phenotype in T cells and dendritic cells (27, 28), and is involved in tumor immune escape (29). Chronic immune stimulation and consecutive IDO activation might also facilitate HIV persistence by inducing T cell apoptosis and tolerance.

IDO1 is the rate-limiting enzyme in the conversion of Trp to Kyn, and thus the Kyn/Trp ratio can be used to estimate IDO1 activity (21, 30). Accelerated Trp breakdown has been reported for several diseases associated with chronic immune activation such as infection, autoimmune syndromes, malignancies, and neurodegenerative disorders, as well as cardiovascular diseases (31). Already in the 90s, accelerated Trp breakdown was shown to correlate with elevated levels of IFN-γ and the immune activation marker neopterin in HIV patients (21–23, 32), and an inflammatory stress-related Trp decrease was suggested to be involved in the development of neuropsychiatric symptoms (33). In 2008, another study demonstrated that HIV-infected patients with depression presented with higher plasma neopterin concentrations, a higher degree of tryptophan degradation, and lower quality of life scores than non-depressive patients (34). Interestingly, correlations between enhanced depression and quality of life scores on the one hand, and tryptophan degradation and neopterin levels on the other hand, were only found in patients without antidepressant medication. In line with these findings, Martinez and colleagues reported recently that depression severity in HIV patients was associated with a decrease in plasma Trp concentration and an increase in Kyn/Trp. In the 12-month follow-up study in a cohort of 504 patients, ART was able to partially reduce Trp breakdown, which went along with improvement of depressive symptoms (35).

## Downstream Metabolites of the Trp-Kyn Pathway

In addition to the depletion of Trp, Kyn downstream products may be involved in immunoregulation and in the pathogenesis of depression. Kyn itself is an endogenous ligand of the arylhydrocarbon receptor and activation of arylhydrocarbon signaling is involved in T-cell differentiation and the development of tolerance (36, 37). 3-hydroxyanthranilic (3-HAA) and quinolinic acid (QA) were shown to selectively induce apoptosis in mouse thymocytes as well as in Th1, but not Th2, cells *in vitro* (38, 39).

Furthermore, a number of kynurenine breakdown products have been reported to be neuroactive. Deregulated production of 3-HAA, 3-hydroxykynurenine (3-HK), and QA has been observed in several neurologic and psychiatric disorders (40).

While some metabolites such as kynurenic acid (KA) can be neuroprotective (41–43), QA released by activated macrophages was shown to exert excitotoxicity (44), thereby contributing to brain injury [see also review by Kandanearatchi and Brew (45)]. When submicromolar concentrations of QA were infused for a longer period into the brain of rats, neuronal loss occurred preferentially in the striatum and the hippocampus – the regions that are involved in HAND neuropathologically (46).

Elevated QA levels were shown in brain tissue of patients with HIV-1-associated dementia (47, 48), and cerebrospinal fluid (CSF) levels of QA were shown to increase during HIV infection and to correlate with HAND severity (49–51). Also, in macaques infected with simian immunodeficiency virus (SIV), the severity of neurological symptoms was related to their CSF QA levels (41). Of note, the HIV-1 proteins Nef and Tat are also able to induce the synthesis of QA by macrophages (52). In a mouse model of peripheral immune activation, which was induced by bacille Calmette-Guerin, IDO activation and upregulation of the quinolinic acid synthesizing enzyme 3-hydroxyanthranilic acid oxygenase (3-HAO) in the brain was shown to play a key role in the development of depressive-like behavior in mice (53). Similarly, a single intracerebroventricular injection of the HIV-1 protein Tat induced depressive-like behavior in exposed mice, and IDO expression was demonstrated in the CNS tissue of the same mice 4 and 24 h after treatment (54). In a recent study on longitudinal changes of Trp-Kyn metabolism in the brains of SIV-infected macaques, Drewes and colleagues confirmed the predictive potential of QA/Trp ratios for CNS associated symptoms (55). Further, while effective combination ART was able to stabilize Trp metabolite concentrations in the CSF of most of these animals, levels could not be restored in the striatum, indicating the only partial effectiveness of the therapy. Interestingly, inhibition of QA formation by 6-chloro-d-tryptophan in HIV-infected macrophages was demonstrated to prevent neuronal damage in human fetal brain aggregate cultures (56).

Furthermore, increased 3-HK levels and elevated kynureninase (KYNU) activity in the frontal cortex of HIV-infected individuals have also been reported, whereby levels were increased in both non-demented and demented HIV patients compared to controls, with the increases being more pronounced for the latter (57).

Accelerated tryptophan breakdown in the periphery and neuropsychiatric changes in the brain are closely linked as brain Kyn metabolite levels are influenced by fluctuations in the concentrations of circulating Trp, Kyn, and 3-HK, which can cross the blood–brain barrier (58). Also, microglial cells and blood-borne cells in the brain can be stimulated to activate the Trp-Kyn pathway during peripheral immune activation (40). Interestingly, IDO activity was significantly increased in the frontal cortex of post-mortem brains of demented HIV-patients compared to age-matched controls, while the activity was only slightly but not significantly elevated in non-demented HIV-patients (57). This finding was consistent with the report of increased enzyme activity in the cerebral cortex of retrovirus-infected macaques (59).

Taken together, the above studies strongly indicate that IDO activation and consecutive accumulation of neurotoxic metabolites play an important role in the development of neurocognitive disturbances. Accordingly, targeting IDO or p38 MAPK expression was proposed by Fu and coworkers as a promising therapy approach to prevent/treat comorbid depressive disorders in HIV-1-infected patients (60).

However, although the conversion to Kyn is the major breakdown route of Trp, accounting for about 90% of Trp metabolism (61, 62), there are also alternative pathways that do not involve the oxidative cleavage of the indole ring, such as the formation of the neurotransmitter serotonin (5-hydroxytryptamine, 5-HT) via tryptophan 5-hydroxylase (TPH, EC:1.14.16.4). Thus, low Trp availability due to persistent immune activation is linked to impaired synthesis of serotonin and can contribute to neurotransmitter imbalance. Indeed, CSF levels of serotonin were found to be significantly lower in a group of 21 asymptomatic, early disease stage HIV-positive patients compared to healthy controls (63). In this study, no correlation was found for 5-HT levels and CD4 cell count or depressive behavior, which could be due to the early stage of disease. In the SIV-infected macaque model, striatal serotonin concentrations decreased during acute and chronic infection and could be partially restored by combined ART, while no correlation of serotonin levels with Trp-Kyn pathway activation marker QA/Trp or encephalitis severity could be observed (55).

The antidepressant paroxetine, which elevates serotonin availability, has previously been applied in different clinical settings with the aim to improve mood and cognitive symptoms (64). Results from patients on IFN-α therapy suggested that cell-mediated immunity and Trp-Kyn pathway activation is not affected by the drug (17). Recently, a combination of fluconazole and paroxetine were shown to be potentially neuroprotective in the SIV-infected macaque model of HIV-associated CNS disease (65). However, although markers of neurodegeneration were decreased in the frontal cortex, serotonin levels could not be significantly elevated and circulating markers of neuroinflammatory origin were not affected. Also, other psychiatric medications are frequently used as adjunct therapies for HIV-associated psychiatric comorbidities and HAND; and trial data on depression and psychosis treatments in HIV-infected patients reported a beneficial effect of selective serotonin reuptake inhibitors (SSRIs) and tricyclic antidepressants (66). However, there are concerns with respect to adverse effects such as worsening of metabolic syndrome when combining antidepressant/antipsychiotic therapies with antiretrovirals (67).

## Vitamin Status, Nutrition, and Inflammatory Stress

Inflammatory disorders are characterized by oxidative stress conditions, which lead to low plasma levels of antioxidants, thus also of vitamins. Nutritional imbalances and deficiencies may contribute to neuropsychiatric disturbances and immune dysregulation associated with HIV-1 infection (68).

For example, low vitamin B_6_ status is related to altered neuropsychiatric function, and the normalization of vitamin B_6_ status in HIV-infected patients was found to be associated with a decline in psychological distress (69). High Trp turnover leads to an increased demand for vitamin B_6_ for downstream processing. Enzymes of the Trp–Kyn pathway that use vitamin B_6_ (pyridoxal 5′-phosphate, PLP) as a cofactor are KYNU, which converts Kyn to anthranilic acid (AA), and 3-HK to 3-HAA, and kynurenine aminotransferases (KAT), which convert Kyn to KA and 3-HK to xanthurenic acid (70). Low vitamin B_6_ status is associated with altered excretion of tryptophan metabolites (71, 72). Also, aromatic-l-amino-acid decarboxylase (AADC), which catalyzes the decarboxylation of l-3,4-dihydroxyphenylalanine (l-DOPA) to dopamine and of 5-hydroxytryptophan (5-HPT) to serotonin, uses PLP as cofactor (73).

Several approaches to influence the synthesis of serotonin and catecholamine neurotransmitters via administration of the appropriate precursor amino acids have been reported (74–76). However, regarding a direct supplementation of tryptophan, it should be considered that there is a risk of elevating concentrations of neurotoxic catabolites (77). Moreover, selective supplementation unlikely results in long-term influence on immunopathogenesis, and thus on the clinical course of cognitive disorders. A multitude of oxidative sensitive molecules are simultaneously affected by inflammatory stress, leading to lower levels of plasma antioxidants (78, 79). Otherwise, more complex dietary interventions might bear a great potential to increase the clinical benefits of HIV patients. Certainly, nutritional and metabolic parameters play an important role in the pathophysiology of HIV infection and might have therapeutic implications (13, 68), which is particularly relevant for individuals suffering from severe nutritional deficiencies (80). For example, in a study cohort of 504 HIV-infected patients undergoing first ART, low protein nutrition was associated with severity of depression (35). It should also be mentioned that about 95% of the body’s serotonin resides in the gut (81) and a number of food-derived antioxidants have been shown to suppress IDO1 activity *in vitro* (82, 83).

In addition to IDO1, hepatic enzyme tryptophan 2,3-dioxygenase (TDO2, EC 1.13.11.11) is able to metabolize Trp. TDO2 activity is regulated by tryptophan levels and by glucocorticoids (84, 85). To relate increased Trp breakdown rates to inflammation-induced IDO1, the concomitant estimation of pro-inflammatory molecules is required. Thereby, the pteridine neopterin has turned out to be a reliable biomarker for inflammation-induced oxidative stress (86).

## Neopterin/Biopterin Metabolism and Inflammatory Stress

Activation of both IDO1 and GCH1 is highly responsive to IFN-γ signaling (87). Neopterin levels are increased in several disorders associated with chronic immune activation (86). In addition, elevated neopterin concentrations are predictive for the development of AIDS and survival in HIV-1 seropositives (23, 33). Also, higher neopterin was shown to correlate with lower levels of plasma antioxidants (79). Of note, the peripheral monocytes of patients with HIV-associated dementia were shown to express lower levels of the antioxidant protein thioredoxin, as well as of enzymes peroxiredoxin and sodium oxide dismutase compared to patients with normal cognition (88). These altered functions of immune cells and the decreased expression of antioxidant defense systems may contribute to inflammation-induced neuronal damage.

Neopterin is formed by the enzyme GTP-cyclohydrolase (GCH1, EC 3.5.4.16). GCH1 converts guanosine-5′-triphosphate (GTP) to 7,8-dihydroneopterintriphosphate, the precursor of neopterin, 7,8-dihydroneopterin, and 5,6,7,8-tetrahydrobiopterin (BH_4_) (86). Most cell types predominantly produce BH_4_ upon activation of GCH1 and lower amounts of 7,8-dihydroneopterin and neopterin. BH_4_ is highly sensitive to oxidation and its concentrations rapidly decrease under oxidative stress conditions (89, 90). In addition, human monocyte-derived macrophages lack sufficient 6-pyruvoyltetrahydropterin synthase (PTPS, EC 4.2.3.12), the enzyme responsible for the formation of BH_4_ from 7,8-dihydroneopterintriphosphate. Therefore, these cells produce large amounts of 7,8-dihydroneopterin/neopterin in a relatively constant ratio of 3:1 (91–93).

BH_4_ is an important cofactor for several monooxygenases including nitric oxide synthase (NOS) (Figure 1). Lack of BH_4_ formation by activated human macrophages may be one reason for the low activity of inducible NOS (iNOS) in immune cells and the generally lower nitrite/nitrate levels in human plasma compared with those in rodents (94).

However, NOS function in other cell types might not be affected in this extent. Interestingly, an increase of serum nitrate in HIV patients has been reported; however, statistical significance of changes differed among these studies depending on disease stage and viral replication and presence of opportunistic infection (95–97). Boven et al. reported elevated levels of nitrotyrosine, indicating the increased formation of peroxynitrite, in brain sections of demented HIV patients compared with non-demented patients (98). The origin of these NOx species is yet unknown. An involvement of neuronal (nNOS) or endothelial NOS (eNOS) in nitric oxide (NO) production could play a role. Importantly, endothelial dysfunction and increased risk of cardiovascular disease are frequently associated with HIV-disease and therapy (99, 100). Of note, NO was shown to strongly inhibit IDO1 activity (101). Both the origin and the functional consequences of changing NO levels in HIV disease have yet to be investigated in more detail.

## BH_4_ Deficiency, Phenylalanine Metabolism, and Catecholamine Synthesis

In addition to being required for NOS activity, BH_4_ is also cofactor for the enzyme phenylalanine 4-hydroxylase (PAH, EC:1.14.16.1). Insufficient cofactor availability impairs the functioning of PAH, resulting in reduced production of catecholamine neurotransmitters dopamine, norepinephrine (noradrenaline), and epinephrine (adrenaline). The precursor amino acid for catecholamines is Tyr, which is formed from Phe by PAH. Also, the conversion of Tyr to l-DOPA via tyrosine 3-monooxygenase (alternative name: tyrosine 3-hydroxylase, TH) requires BH_4_ as cofactor (92).

Dysregulation of Phe metabolism was found in patients with cancer, patients with multiple trauma with sepsis, and in the elderly, as well as in patients with HIV-1 infection (19, 20, 102–104). Plasma Phe/Tyr ratio measured in HIV-1-infected individuals was found to be increased and to correlate with the concentrations of the immune activation marker neopterin, as well as with HIV-RNA levels and CD4+ counts (20). In the same study, it was shown that effective ART could reduce plasma concentrations of Phe and neopterin. The Phe/Tyr ratio is a convenient way to determine PAH activity in the absence of a reliable methodology for BH_4_ measurements (105). Thus, the increase of Phe/Tyr ratio found in HIV-1-infected individuals is a strong indication of BH_4_ deficiency, which is most probably due to oxidative loss of the oxidation-sensitive cofactor in the state of chronic immune activation.

Changes in dopaminergic neurotransmission have been reported for HIV-infection in a number of studies. Decreased availability of dopamine in the central nervous system is correlated with low performance in neuropsychological functions and cannot be fully rescued by highly active ART (HAART) treatment (106, 107). Also, increased dopamine catabolism can contribute to dopamine deficiency. Monoamine oxidase activity was reported to be enhanced in specific brain regions of HIV-infected individuals and this was associated with HIV encephalitis (108).

Of note, the activity of the enzyme involved in serotonin synthesis, TPH, is also dependent on BH_4_ levels, suggesting that potential decreases in BH_4_ during HIV infection may contribute to the losses in serotonin levels as well as to the dysregulation of dopamine and other catecholamine neurotransmitters.

## Metabolomic and Proteomic Approaches for Biomarker Identification

In recent times, a number of new metabolites with potential prognostic value have been identified in large-scale metabolome biofluid analysis of HIV patients (109). Importantly, several studies confirmed the changes in metabolism of the above-mentioned amino acids Trp, Phe, and Tyr.

By analyzing the profile of oral metabolites in HIV-infected individuals, including ART-experienced and ART-naïve patients, in comparison to healthy controls, Ghannoum et al. reported differences in levels of metabolites that belong to especially carbohydrate and amino acid metabolism (110). In this study, Trp, Phe, and Tyr, as well as several downstream metabolites of these amino acids, were found to be upregulated in HIV-infected individuals compared to controls. Both Trp and Phe concentrations were somewhat higher in the oral wash of therapy-naïve versus experienced individuals. In line with previous results (20), the Phe/Tyr ratio was a reliable marker for the monitoring of the immune status during infection in this study. Otherwise, while in the circulation Trp breakdown via the increased activity of IDO1 in immune cells is highly accelerated (21), the consistently elevated levels of Trp in the oral wash samples indicate distinct metabolic properties in the oral cavity (110). Oral wash is an interesting body fluid for non-invasive diagnostics, but serum or plasma may still represent a better alternative when investigating immunological parameters. Although less accessible, CSF, which is close to the site of inflammation and linked to the neuropathology, may be best suited for assessing the association of various molecules in particular with disease-associated central nervous system-related symptoms (111, 112). A recent study assessing CSF metabolites in HIV patients with neurocognitive impairments reported alterations in Kyn levels, however, only for patients not on ART (113). In the same study, Phe and Tyr metabolism associated metabolite sets were enriched in patients with cognitive impairments. Besides oxidative stress and mitochondrial function, metabolite profiles revealed aging-related pathways to be associated with neurocognitive deficits.

Using a proteomic approach, Laspiur et al. (114) identified several molecules that are uniquely present in CSF of HIV patients with cognitive impairments. Among those molecules were sodium oxide dismutase, an antioxidant enzyme that was previously reported to be upregulated in the brains of HIV-demented patients compared to control patients (98), and migration inhibitory factor-related protein 14 (also known as protein S100A9), which is involved in the regulation of inflammatory processes and the immune response (114). In addition, other proteome studies have identified potential markers that might be associated with neuronal damage and inflammation (115, 116).

## Therapeutics and Future Directions

A number of *in vitro* and animal studies are currently evaluating the potential of targeting IDO to limit HIV infection in combination with antiretroviral treatment. Blockade of IDO by 1-methyl-d-tryptophan reduced viral loads in the plasma and lymph nodes of SIV-infected macaques that were also treated with ART (117). Whether long-term treatment with this combination is well tolerated, and whether it is effective at preventing neuronal damage or depression and/or at facilitating immune reconstitution still needs to be investigated. In fact, in animal tumor models, IDO1 inhibition by chemical or genetic interventions has been associated with the (re)activation of therapeutically relevant anticancer immune responses (118). Similar effects could be beneficial for immunodeficient patients.

Other treatment options are also currently being investigated. In a pilot study involving treatment of four HIV infected patients, high dose nicotinamide was effective at increasing tryptophan concentrations of patients (119). In addition, Lebouche and colleagues are investigating the effects of combined ART and niacin treatment on neurocognition and immune status, as well as on lipid metabolism of HIV-infected patients (120).

Recently, the combination of fluconazole and paroxetine (FluPar) was suggested as a therapeutic strategy for the treatment/prevention of neurological damage in HIV-infected patients. FluPar was shown to be protective against HIV gp120- and Tat-mediated neurotoxicity in a macaque model of SIV infection. Also, treatment of patients with ART and paroxetine (or another SSRI) was proposed as adjunctive neuroprotective and neuroregenerative therapy to treat HIV-infected individuals (121).

The CCR5 inhibitor maraviroc was shown to inhibit CNS replication of SIV in infected macaques, as well as to lower monocyte and macrophage activation, and to exert neuroprotective effects (122). In a study with 15 HIV-infected individuals, this drug improved the neurocognitive performance of the patients (123).

In a recent study in Wistar rats, facilitated transport of efavirenz (a non-nucleoside reverse transcriptase inhibitor) across the blood–brain barrier using phenylalanine-anchored solid lipid nanoparticles was shown to improve bioavailability and maintain therapeutic levels in the brain for an extended period of time, probably enabling a significant eradication of the viral load (124).

However, as discussed in Section “Vitamin Status, Nutrition, and Inflammatory Stress,” nutritional interventions supporting drug therapy might be a first and very effective step to personalized treatment with less side effects.

## Conclusion

A major issue in the treatment of HIV/AIDS is the development of neurocognitive disturbances in individuals despite effective therapy, although symptoms develop in a milder form or with changed dynamics (4, 10, 125). Several factors are suggested to be involved in the progression of HAND, including the inability of drugs to effectively cross the blood–brain barrier, the development of resistant viral strains, and neuronal toxicity induced by viral proteins or chronic inflammation.

Elevations in Phe/Tyr, Kyn/Trp, and neopterin levels were shown in patients with HIV-1 infection, with the levels of neopterin in HIV-1 seropositives providing predictive information for the progression of AIDS and survival (20, 23). The cellular immune system is activated during HIV-1 infection, which leads to the activation of IFN-γ-dependent pathways including neopterin production via GCHI and tryptophan catabolism via IDO1. The concentrations of these biomarkers have been found to be altered in mental disorders and in disease-associated mood disturbances, and to correlate with depressive symptoms, especially, in long-lasting chronic diseases (31, 126). Of note, higher neopterin and Kyn/Trp were demonstrated in patients with HIV-infection (34) and accelerated Trp breakdown was shown to correlate with neuropsychiatric symptoms in HIV-patients (21, 33). In addition, chronic immune activation correlates with the decreased turnover of Phe (19), which has also been reported in HIV infection (20). Tyr and Trp are important precursors of serotonin and the dopaminergic neurotransmitters. The increased production of neopterin at the expense of BH_4_ and the lability of BH_4_ molecule under oxidative stress conditions (92) provides a rational link for the decreased activity of TPH and PAH enzymes, which use BH_4_ as a cofactor.

Recent metabolome and proteome analyses of changes occurring in the course of HIV-1 infection and therapy could confirm the dysregulation of amino acid metabolism and identify further pathways and molecules involved in HIV-associated neuroinflammation.

Numerous studies have reported an association between changes in immunologic parameters, such as the impaired activities of immunocompetent cells or increases in inflammatory mediators, and cytokine production with the activation of neuroendocrine pathways, altered neurotransmitter metabolism, and thus changes in behavior (17, 127). Current clinical parameters used to guide HIV-treatment are viral load and CD4 counts. These can probably deliver more diagnostic information when used in combination with an estimation of amino acid levels in patient serum. The targeted measurement of the well-established biomarkers neopterin, Kyn/Trp, and Phe/Tyr could be used to monitor the course of disease and judge the effectiveness of treatments in a reliable and relatively cost-effective manner. Although it was reported that ART could beneficially influence Phe/Tyr (20) and Kyn/Trp ratios (127), it still remains to be investigated whether a therapy-induced stabilization of amino acid levels also improves mood and quality of life in patients.

## Concluding Remarks

Changes in mood status can be observed in the very early stages of HIV-1 infection (5) and, notwithstanding ART, HIV-1-infected individuals may develop cognitive impairment (10). The molecular changes underlying these infection-associated disturbances have not yet been characterized. Changes in the metabolism of the amino acid tryptophan and phenylalanine have been associated with HIV disease and were of predictive value. Both amino acids are precursors for neurotransmitter biosynthesis, providing a link to the development of disease-associated neurocognitive impairments. Therefore, monitoring amino acid metabolism in HIV-1-infected patients during ART could be useful in adapting personalized treatment regimens.

## Conflict of Interest Statement

The authors declare that the research was conducted in the absence of any commercial or financial relationships that could be construed as a potential conflict of interest.
